# Acute liver failure in patients admitted to the intensive care unit—a Viennese retrospective single-center analysis

**DOI:** 10.1007/s00508-025-02539-1

**Published:** 2025-05-26

**Authors:** Patrick Haselwanter, Seanna Fairfield, Marlene Riedl-Wewalka, Monika Schmid, Albert Friedrich Stättermayer, Thomas Reiberger, Michael Trauner, Christian Zauner, Mathias Schneeweiss-Gleixner

**Affiliations:** Department of Medicine III, Division of Gastroenterology and Hepatology, Intensive Care Unit 13H1, Medical University of Vienna, Vienna, Austria

**Keywords:** Liver failure, Liver transplantation, Critical care, Hepatology, Epidemiology

## Abstract

**Background:**

Acute liver failure (ALF) is characterized by a rapid deterioration of liver function and a high mortality without transplantation depending on etiology and onset. Immediate transfer to a dedicated intensive care unit (ICU) and evaluation for high-urgency liver transplantation (HU-LTx) is recommended to maximize chances of survival. Data on ALF epidemiology are limited, particularly for Central Europe.

**Methods:**

This retrospective single-center study included all ALF patients admitted to the ICU of the Department of Gastroenterology and Hepatology at the Vienna General Hospital between 2012 and 2024.

**Results:**

Overall, 31 patients (median age of 44 [interquartile range, IQR 32–56] years, 20 [65%] female) were included. The primary causes of ALF were viral infections (*n* = 8; 26%), autoimmune hepatitis (*n* = 5; 16%), drug-induced liver injury (DILI; *n* = 3; 10%), and Wilson’s disease (*n* = 4; 13%), while in 8 patients (26%) no cause was identified. Median length of ICU stay was 12 (IQR 4–21) days, with mean sequential organ failure assessment (SOFA) and simplified acute physiology score II (SAPS II) scores of 10.55 ± 4.56 and 40.97 ± 14.84. Overall ICU survival was 61% (*n* = 19). Non-HU-LTx patients (*n* = 18) had an ICU survival of 44%. HU-LTx was performed in 13 patients (42%), with 12 patients (92%) surviving 28 days. The 6‑month overall survival of HU-LTx patients was 85%.

**Conclusion:**

The diverse causes of ALF in Central Europe include most commonly viral infections, autoimmune hepatitis, and DILI. HU-LTx was required and performed in almost half of patients and was associated with favorable survival rates, underscoring the importance of ICU management and early transfer to liver transplantation centers in the management of ALF.

**Supplementary Information:**

The online version of this article (10.1007/s00508-025-02539-1) contains supplementary material, which is available to authorized users.

## Introduction

Acute liver failure (ALF) represents a rapidly developing syndrome caused by severe damage of the liver, leading to the inability to perform its normal synthetic, metabolic, and endo-/exocrine functions [1]. The European Association for the Study of the Liver (EASL) defines ALF as an initial severe acute liver injury (ALI) characterized by a 2–3× upper limit of norm (ULN) elevation of transaminases and an impaired liver function in patients without pre-existing chronic liver diseases (i.e., international normalized ratio [INR] > 1.5 and overt hepatic encephalopathy [HE]) [2]. Necrosis and apoptosis of hepatocytes lead to a continuous release of proinflammatory cytokines, chemokines, and reactive oxygen species, resulting in multiorgan failure and cerebral edema with consecutive poor outcomes [3, 4]. Besides specific treatment of the triggering cause of ALF, an immediate transfer to a tertiary center and evaluation for high-urgency liver transplantation (HU-LTx) is crucial to improve survival in ALF patients [2].

A broad spectrum of ALF etiologies needs to be considered, which differ globally and regionally in prevalence, depending on hygienic standards, drug use patterns, and vaccination status. In general, ALF is more frequent in developing countries [2, 5]. While virally induced liver failure dominates in Asian countries due to the lack of nationwide vaccination programs, acetaminophen-induced liver failure is the most common cause of ALF in the United Kingdom and the United States due to unrestricted drug policies [5–8]. In Europe, drug-induced liver injury (DILI) as a burden for ALF has been reported more frequently, with increasing incidence over the last two decades [5, 9]. Autoimmune hepatitis (AIH), Wilson’s disease, vascular disorders (i.e., Budd–Chiari syndrome), pregnancy-related ALF, mushroom poisoning, hemophagocytic lymphohistiocytosis (HLH), and malignancies represent additional, but less frequent etiologies [5].

Due to the lack of centralized data collection, the total burden of ALF and the distribution of its etiologies in Europe are still unknown. Therefore, we conducted a retrospective explorative study to investigate patients with ALF treated at a specialized intensive care unit (ICU) of a large tertiary center at the Vienna General Hospital. Within the scope of this study, we delineated etiologies, clinical features, outcome parameters, and laboratory trajectories of an ALF patient cohort from a large tertiary center in Central Europe.

## Patients, materials, and methods

### Study design and setting

We conducted a retrospective explorative study of patients admitted to the ICU of the Department of Gastroenterology and Hepatology at the Vienna General Hospital, a large LTx center in Eastern Austria. All patients over 18 years with ALF treated at the ICU from January 1, 2012, to January 1, 2024, were included in this study. ALF was defined according to the current EASL definition [2]. Notably, patients with chronic liver disease and/or acute on chronic liver failure were not included in this study. Patients with an ALI or anticipated ALF who did not fulfill the EASL criteria of ALF were also excluded [2]. Detailed information on the inclusion process of our study population is depicted in the flowchart of Supplemental Figure S1. According to the onset of ALF (development from jaundice to HE), patients were separated into hyperacute (0–7 days), acute (8–28 days), or subacute (28 days to 12 weeks) liver failure [10]. The observation period was set from ICU admission to ICU discharge or death. We analyzed clinical and epidemiological characteristics, laboratory parameters, ICU- and ALF-specific parameters, and outcome parameters for this study period.

The study was conducted under the principles of the Declaration of Helsinki, including current revisions and the rules of Good Clinical Practice of the European Commission [11]. It was approved by the local ethics committee of the Medical University of Vienna (ethics vote number: 1229/2024). Due to the retrospective study design, the local ethics committee waived the informed consent requirement.

### Data collection

Data were extracted from electronic patients’ charts (IntelliSpace Critical Care and Anesthesia, Philips, Amsterdam, The Netherlands) of all patients admitted to the ICU. These electronic patient charts are used for routine documentation at all ICUs at the Medical University of Vienna. Thereby, data are documented prospectively, including precise information on patient characteristics, vital signs, laboratory parameters, medication, and ICU-specific therapies, including invasive mechanical ventilation (IMV), renal replacement therapy (RRT), and other extracorporeal assist devices. Moreover, data on HU-LTx and time of transplantation were also collected. Simplified acute physiology score II (SAPS II) and sequential organ failure assessment score (SOFA) were used to calculate disease severity within the first 24 h of ICU admission [12, 13].

### Statistical analysis

The primary objective of this study was to provide a demographic overview of ALF patients treated at an ICU in a single center in Central Europe. We focused on clinical and epidemiological characteristics, as well as laboratory, ICU-specific, ALF-specific, and outcome parameters during their respective ICU stays.

In the case of normal distribution, quantitative parameters are presented as mean ± standard deviation (SD), otherwise as median (interquartile range [IQR]). Normal distribution was tested with the Shapiro–Wilk test with a *p*-value > 0.05. Qualitative parameters are reported as absolute numbers (relative proportions in %). Categorical variables were compared using Pearson’s Chi-square test. The t‑test was employed to compare metric variables between groups if the data followed a normal distribution. Otherwise, the Mann–Whitney U test was used for nonparametric variables. The Wilcoxon test was used for nonparametric variables to compare median changes of laboratory parameters. The probability of ICU survival was calculated using Kaplan and Meier’s product limit method. Differences concerning ICU survival among subgroups (patients with HU-LTx and without HU-LTx) were determined by log-rank test. The statistical analyses were performed with GraphPad Prism 10 (GraphPad Software, San Diego, CA, USA) and IBM SPSS (version 28, IBM, Armonk, NY, USA).

## Results

### Demographics and clinical characteristics

**Fig. 1:**
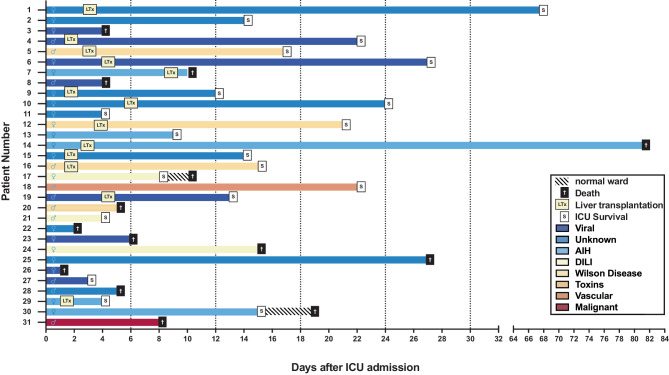
Clinical characteristics and timeline of acute liver failure (ALF) patients admitted to the ICU. Individual patient courses refer to the numbers in Table 2. Patients receiving high-urgency liver transplantation (HU-LTx) were highlighted with “LTx” in a yellow square. Intensive care unit (ICU) survival is marked with an “S” in a white square, whereas patients’ death is marked with a cross in a black square. ICU-length of stay (LOS) with ALF etiologies were given in bars with different colors. If patients died within 30 days in a normal ward, the duration at the normal ward was given in bars with oblique black stripes. *AIH* autoimmune hepatitis, *ALF* acute liver failure, *DILI* drug-induced liver injury, *ICU* intensive care unit, *HU-LTx* high-urgency liver transplantation, *LOS* length of stay

**Fig. 2:**
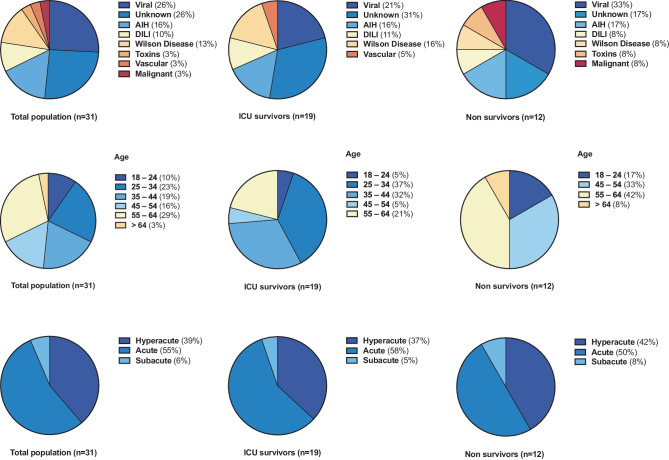
Proportional representation of acute liver failure (ALF) etiologies in total population, intensive care unit (ICU) survivors and nonsurvivors. ALF etiologies are given in different colors, as shown in Fig. 2. Relative frequencies of etiologies in the total population, ICU survivors, and nonsurvivors are also depicted in Table 1. *AIH* Autoimmune Hepatitis, *ALF* acute liver failure, *DILI* drug-induced liver injury, *ICU* intensive care unit

In total, 31 adult patients (20 women, 65%, and 11 men, 35%) with ALF admitted to a specialized ICU at a large tertiary center in Central Europe were included and analyzed in our study. Patients’ baseline and individual characteristics are provided in Tables 1 and 2. Detailed information on the individual clinical course of ALF after ICU admission is depicted in Fig. 1. The patients’ median age was 44 (32–56) years. The most frequent cause of ALF was viral infection (*n* = 8, 26%). In 5 patients, ALF was related to AIH (16%), and in 4 patients to Wilson’s disease (13%). DILI (*n* = 3, 10%; *n* = 1 chemotherapy and *n* = 2 idiosyncratic), toxins (*n* = 1, 3%; amanita phalloides), vascular diseases (*n* = 1, 3%; hypoxic hepatitis), and malignant diseases (*n* = 1, 3%; upper gastrointestinal adenocarcinoma with liver infiltration) were relatively rare. In 8 patients (26%), the etiology of ALF remained unknown. Most patients presented with acute (*n* = 17, 55%) or hyperacute (*n* = 12, 39%) liver failure, whereas cases of subacute liver failure were the minority (*n* = 2, 6%). The proportional distribution of etiology, age, and onset of liver failure is illustrated in Fig. 2 and Supplemental Figure S2.

**Table 1:** Comparison of baseline characteristics between ICU survivors and nonsurvivors

	Total population	ICU survivors	Nonsurvivors	*p‑value*
***N* (%)**	31 (100)	19 (61)	12 (39)	–
**Age, median (IQR)**	44 (32–56)	39 (32–48)	55 (47–60)	0.0526
**Age distribution**				
*18–24, n (%)*	3 (10)	1 (5)	2 (17)	0.2955
*25–34, n (%)*	7 (23)	7 (37)	0	*0.0168*
*35–44, n (%)*	6 (19)	6 (32)	0	*0.0301*
*45–54, n (%)*	5 (16)	1 (5)	4 (33)	*0.0384*
*55–64, n (%)*	9 (29)	4 (21)	5 (42)	0.2180
*>* *64, n (%)*	1 (3)	0	1 (8)	0.2008
**Male/female**	11/20	7/12	4/8	0.8423
**BMI, median (IQR)**	25.7 (22.2–28.6)	24.2 (22–26.5)	27.8 (24.3–35.8)	0.0506
**Etiology of ALF**				
*Viral, n (%)*	8 (26)	4 (21)	4 (33)	0.4465
HBV, *n* (%)	6 (75)	3 (75)	3 (75)	0.5272
HAV, *n* (%)	1 (12.5)	1 (25)	0	0.4191
EBV, *n* (%)	1 (12.5)	0	1 (25)	0.2008
*Unknown, n (%)*	8 (26)	6 (31)	2 (17)	0.3553
*AIH, n (%)*	5 (16)	3 (16)	2 (17)	0.9484
*DILI, n (%)*	3 (10)	2 (11)	1 (8)	0.8405
*Wilson Disease, n (%)*	4 (13)	3 (16)	1 (8)	0.5463
*Toxins, n (%)*	1 (3)	0	1 (8)	0.2008
*Vascular, n (%)*	1 (3)	1 (5)	0	0.4191
*Malignant, n (%)*	1 (3)	0	1 (8)	0.2008
**ICU LOS (days), median (IQR)**	12 (4–21)	14 (8.5–21.5)	5.5 (4–11.3)	0.1126
**Vasopressor therapy, *n* (%)**	21 (68)	11 (58)	10 (83)	0.1399
**IMV, *n* (%)**	26 (83)	15 (78)	11 (92)	0.3483
**Reason for IMV**				
*HE, n (%)*	16 (64)	7 (47)	9 (75)	*0.0383*
*HU-LTx, n (%)*	7 (28)	7 (47)	0	*0.0168*
*Respiratory failure, n (%)*	3 (12)	1 (7)	2 (25)	0.2955
**Length of IMV (days), median (IQR)**	2 (1–6)	5 (1.5–8)	2 (1–5.5)	0.9522
**ICU adm. to IMV (days), median (IQR)**	1 (0–3)	2 (0–2.5)	1 (0.5–3.5)	0.7988
**EC therapy**^**a**^**, *n* (%)**	24 (77)	14 (74)	10 (83)	0.5314
**RRT, *n* (%)**	23 (74)	13 (68)	10 (83)	0.3553
**ECMO, *n* (%)**	2 (6)	1 (5)	1 (8)	0.7346
**PE, *n* (%)**	2 (6)	1 (5)	1 (8)	0.7346
**MARS, *n* (%)**	5 (16)	3 (5)	2 (17)	0.9484
**CytoSorb adsorbers, *n* (%)**	9 (29)	7 (37)	2 (16)	0.2280
**SAPS II**^**b**^**, mean** **±** **SD**	40.97 ± 14.84	38.05 ± 11.92	45.58 ± 18.18	0.1728
**SOFA**^**b**^**, mean** **±** **SD**	10.55 ± 4.56	10.05 ± 5.16	11.33 ± 3.47	0.4555
**Liver transplant, *n* (%)**	13 (42)	11 (58)	2 (17)	*0.0234*
**28-day survival, *n* (%)**	18 (58)	17 (89)	1 (8)	*<* *0.0001*
**3‑month survival, *n* (%)**	17 (55)	17 (89)	0	*<* *0.0001*
**6‑month survival, *n* (%)**	17 (55)	17 (89)	0	*<* *0.0001*

*AIH* autoimmune hepatitis; *ALF* acute liver failure; *BMI* body mass index; *DILI* drug-induced liver injury; *EBV* Epstein–Barr virus; *EC* extracorporeal; *ECMO* extracorporeal membrane oxygenation; *HAV* Hepatitis A virus; *HBV* Hepatitis B virus; *HE* hepatic encephalopathy; *HU*-*LTx* high-urgency liver transplantation; *ICU LOS* intensive care unit length of stay; *IMV* invasive mechanical ventilation; *IQR* interquartile range; *n* population size; *PE* plasma exchange; *RRT* renal replacement therapy; *SAPS II* simplified acute physiology score II; *SD* standard deviation; *SOFA* sequential organ failure assessment score

^a^EC Therapy including RRT, plasma exchange, liver-assist devices, ECMO

^b^SAPSII and SOFA score were calculated within the first 24 h after admission

**Table 2:** Patients’ characteristics

Pt. *n*	Age	m/f	ALF etiology	ALF type	SOFA score^a^	SAPS II Score^a^	HE^b^ admission	HE^b^ peak	IMV^c^	Reason for IMV	VP^c^	EC therapy^d^	HU-LTx	HU-LTx listed	Reason for no HU-LTx	ICU survival	28-day survival	3‑month survival	Reason for death
1	34	f	UNK	Acute	4	22	1	4	Yes	HE	Yes	Yes	Yes	Yes	–	Yes	Yes	Yes	–
2	33	f	UNK	Acute	4	28	2	2	No	–	No	Yes	No	Yes	Not needed	Yes	Yes	Yes	–
3	47	f	Viral	Acute	9	33	3	4	Yes	HE	Yes	Yes	No	Yes	Death before LTx	No	No	No	MOF
4	43	m	Viral	Hyperacute	5	36	1	2	Yes	LTx	Yes	Yes	Yes	Yes	–	Yes	Yes	Yes	–
5	22	m	Wilson	Acute	14	27	2	3	Yes	LTx	Yes	Yes	Yes	Yes	–	Yes	Yes	Yes	–
6	29	f	Viral	Hyperacute	16	41	4	4	Yes	HE	Yes	Yes	Yes	Yes	–	Yes	Yes	Yes	–
7	18	f	AIH	Acute	5	20	2	4	Yes	HE	Yes	Yes	Yes	Yes	–	No	No	No	MOF
8	62	m	Viral	Acute	8	50	2	4	Yes	HE	Yes	No	No	Yes	Death before LTx	No	No	No	MOF
9	38	f	UNK	Acute	10	31	2	4	Yes	HE	No	No	Yes	Yes	–	Yes	Yes	Yes	–
10	44	f	UNK	Acute	5	42	4	4	Yes	HE	Yes	Yes	Yes	Yes	–	Yes	Yes	Yes	–
11	58	f	UNK	Hyperacute	15	47	4	4	Yes	HE	Yes	Yes	No	No	Not needed	Yes	Yes	Yes	–
12	42	f	Wilson	Acute	9	41	1	2	Yes	LTx	Yes	Yes	Yes	Yes	–	Yes	Yes	Yes	–
13	32	f	AIH	Hyperacute	8	34	4	4	Yes	HE	No	Yes	No	No	Not needed	Yes	Yes	Yes	–
14	54	f	AIH	Acute	9	32	2	4	Yes	HE	Yes	Yes	Yes	Yes	–	No	Yes	No	ICB
15	42	f	UNK	Acute	18	48	2	2	Yes	LTx	Yes	Yes	Yes	Yes	–	Yes	Yes	Yes	–
16	32	m	Wilson	Hyperacute	14	43	1	1	Yes	LTx	Yes	Yes	Yes	Yes	–	Yes	Yes	Yes	–
17	27	f	DILI	Acute	7	21	3	3	No	–	No	Yes	No	No	Active IVDA	Yes	No	No	CV
18	57	m	Vascular	Acute	4	45	3	4	Yes	HE	Yes	No	No	No	Not needed	Yes	Yes	Yes	–
19	52	m	Viral	Acute	13	42	2	3	Yes	LTx	No	No	Yes	Yes	–	Yes	Yes	Yes	–
20	22	m	Wilson	Hyperacute	14	36	4	4	Yes	RF	Yes	Yes	No	Yes	Death before LTx	No	No	No	MOF
21	58	m	DILI	Hyperacute	8	49	3	3	No	–	No	No	No	No	Not needed	Yes	Yes	Yes	–
22	55	f	Toxin	Hyperacute	12	44	4	4	Yes	HE	Yes	Yes	No	Yes	Death before LTx	No	No	No	MOF
23	70	f	Viral	Hyperacute	17	69	4	4	Yes	HE	Yes	Yes	No	No	High age	No	No	No	MOF
24	59	f	DILI	Acute	14	79	4	4	Yes	HE	Yes	Yes	No	No	Malignancy	No	No	No	MOF
25	52	f	UNK	Subacute	13	51	4	4	Yes	RF	Yes	Yes	No	No	Malignancy	No	No	No	MOF
26	45	f	Viral	Hyperacute	14	58	1	4	Yes	HE	Yes	Yes	No	Yes	Death before LTx	No	No	No	MOF
27	39	m	Viral	Hyperacute	10	34	3	4	No	–	No	Yes	No	No	Not needed	Yes	Yes	Yes	–
28	56	m	UNK	Hyperacute	13	54	3	4	Yes	HE	No	Yes	No	No	Malignancy	No	No	No	MOF
29	28	f	AIH	Acute	6	22	3	3	Yes	LTx	No	No	Yes	Yes	–	Yes	Yes	Yes	–
30	55	f	AIH	Subacute	21	70	4	4	Yes	RF	Yes	Yes	No	No	Not needed	Yes	No	No	UNK
31	62	m	Malignant	Acute	8	21	2	4	No	–	No	No	No	No	Malignancy	No	No	No	MOF

*AIH* autoimmune hepatitis; *ALF* acute liver failure; *CV* cardiovascular; *DILI* drug-induced liver failure; *EC* extracorporeal; *f* female; *HE* hepatic encephalopathy; *HU-LTx* high-urgency liver transplantation; *ICB* intracranial bleeding; *ICU* intensive care unit; *IMV* invasive mechanical ventilation; *IVDA* intravenous drug abuse; *MOF* multiple organ failure; *m* male; *Pt.* patient; *RF* respiratory failure; *SAPS II* simplified acute physiology score II; *SOFA* sequential organ failure assessment score; *UNK* unknown; *VP* vasopressor

^a^SAPS II and SOFA score were calculated within the first 24 h after ICU admission

^b^According to West Haven criteria

^c^IMV and Vasopressor therapy during ICU stay

^d^EC Therapy including renal replacement therapy, plasma exchange, liver-assist devices, extracorporeal membrane oxygenation

### ICU-specific management

The median ICU length of stay (LOS) was 12 days (4–21), and the mean SAPS II and SOFA scores were 40.97 ± 14.84 and 10.55 ± 4.56, respectively. The overall ICU survival was 61% (*n* = 19). Invasive mechanical ventilation (IMV) was required in 83% of patients (*n* = 26), with a median length of IMV of 2 days (1–6) and a median time from admission to intubation of 1 day (0–3). The most common reason for intubation was neurological deterioration due to HE (*n* = 16, 64%), followed by elective intubation for HU-LTx (*n* = 7, 28%) or respiratory failure (*n* = 3, 12%). Moreover, 68% of ALF patients (*n* = 21) needed noradrenalin support, of which 57% required the addition of vasopressin. The median peak dose for noradrenaline was 0.417 µg/kg/min (0.196–0.917 µg/kg/min), and the median peak dose for vasopressin was 2 I.U./h (2–2.25 I.U./h). Extracorporeal therapy was established in 24 patients (77%), with RRT in 23 cases (74%). Other extracorporeal devices, such as extracorporeal membrane oxygenation (*n* = 2, 6%) plasma exchange (*n* = 2, 6%), and MARS (*n* = 5, 16%), were less frequently applied. Additional hemadsorptive therapy with CytoSorb filter was conducted in 9 patients (29%).

### ICU survivors vs. ICU nonsurvivors

Differences between ICU survivors and nonsurvivors are displayed in Table 1. Except for median peak noradrenaline support, there were no statistically significant differences between ICU survivors and nonsurvivors. By trend, ICU survivors were younger (39 vs. 55 years, *p* = 0.0526) with longer median ICU-LOS (14 vs. 5.5 days, *p* = 0.1126) and lower mean SAPS II (38.05 vs. 45.58, *p* = 0.1728) and SOFA scores (10.05 vs. 11.33, *p* = 0.4555). Vasopressors were more frequently used in nonsurvivors (83% vs. 58%, *p* = 0.1399) with significantly higher median noradrenalin peak doses (0.916 vs. 0.196 µg/kg/min, *p* = 0.0007).

### High-urgency liver transplantation

**Fig. 3:**
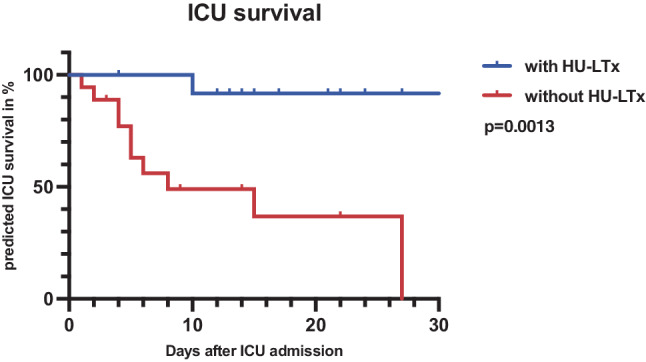
Predicted intensive care unit (ICU) survival of acute liver failure (ALF) patients with high-urgency liver transplantation (HU-LTx) and without HU-LTx. All included patients were separated into patients with HU-LTx after ICU admission, highlighted as a blue line, and patients without HU-LTx, highlighted as a red line. ICU survival of both groups was calculated with a Kaplan–Meier-based analysis, where patients with HU-LTx were associated with a statistically significant increase in predicted survival compared to patients without HU-LTx (*p* = 0.0013 by log-rank test). *ALF* acute liver failure, *ICU* intensive care unit, *HU-LTx* high-urgency liver transplantation

HU-LTx was conducted in 42% of included patients (*n* = 13) with a median time from admission to the transplant of 3 days (2–4). All patients were evaluated for a potential HU-LTx. A total of 19 patients were listed (*n* = 13 transplanted, *n* = 5 died before potential transplantation, and *n* = 1 did not need an HU-LTx), and 12 were not listed. Six of those were rejected for HU-LTx due to malignancy (*n* = 4), active intravenous drug abuse (IVDA; *n* = 1), and high age (*n* = 1), and further 6 patients could be delisted and did not need an HU-LTx due to an amelioration of liver function. As expected, HU-LTx had a significant impact on ICU-, 28-day-, 3‑month, and 6‑month survival, as seen in the Kaplan–Meier analysis on ICU survival in Fig. 3 (*p* = 0.0013). Further details of baseline characteristics among the HU-LTx subgroups are depicted in Supplemental Table S1.

### Gender-specific differences

Gender-specific differences in our patient population are highlighted in Supplemental Table S2. We observed statistically significant differences in extracorporeal therapy (*n* = 18 vs. *n* = 6, *p* = 0.0238), RRT (*n* = 18 vs. *n* = 5, *p* = 0.0066), and length of IMV (5.5 vs. 1 day, *p* = 0.0395) between women and men. By trend, the etiology of ALF was more frequently unknown (*n* = 7 vs. *n* = 1, *p* = 0.1147) or related to AIH (*n* = 5 vs. *n* = 0, *p* = 0.0701) in females, whereas ALF in men was more often related to Wilson disease (*n* = 3 vs. *n* = 1, *p* = 0.0767).

### Trajectory of laboratory parameters

Details on the changes in laboratory parameters over the course of the ICU stay are depicted in Supplemental Table S3 and Supplemental Figures S3 and S4. As expected, liver chemistries indicating liver cell damage and impaired detoxification capacity (i.e., ASAT, ALAT, bilirubin, alkaline phosphatase [AP], and γ‑glutamyl transferase [γ-GT]) were initially highly elevated. A significant decrease in all mentioned parameters was observed 24 h after admission. Simultaneously, prothrombin time and INR, reflecting impaired synthesis of coagulation parameters, were deteriorated, especially at admission and after 24 h. Of note, ammonia levels, representing a potential marker for neurological deterioration, were elevated (85.7 µg/dL [63.1–105.9]) and slightly increased 24 h after admission (100.9 µg/dL [60.2–124.6], *p* = 0.5390) [14]. Routine inflammation parameters (i.e., C‑reactive protein [CRP] and white blood count [WBC]) remained low within the first 24 h after ICU admission but slightly increased until discharge or death.

There were no differences regarding ICU survival in liver chemistries or inflammation parameters at ICU admission and after 24 h. AP and γ‑GT significantly increased in ICU survivors (*p* = 0.0085 and *p* = 0.0181). As expected, bilirubin significantly decreased among survivors at ICU discharge (*p* < 0.0001). Prothrombin time, fibrinogen, and INR showed significant improvements among survivors until discharge (*p* < 0.0001, *p* = 0.0002, and *p* = 0.0010), resembling a normalization of coagulation. Interestingly, there was a trend towards lower platelet counts (193 vs. 118.5 G/L, *p* = 0.3014) and hemoglobin (12.2 vs. 10.75 G/L, *p* = 0.5155) at admission among nonsurvivors, which remained until discharge or death.

## Discussion

In current clinical practice, ALF still represents a highly complex disease requiring specialized intensive care treatment and early evaluation for HU-LTx. Within the scope of this single-center study, we delineated the etiologies, clinical features, outcome parameters, and laboratory trajectories among patients admitted to the ICU due to ALF at a large tertiary center in Eastern Austria over the last decade.

The proportional distribution of ALF causes in our cohort mostly resembled that of other European studies. With an incidence of 26%, viral infection was the most common cause in our study population, which is comparable with published data from other European countries (incidence rates ranging from 7 to 37%) [15–18]. Although there is a significant trend of declining cases of viral-induced ALF among Western countries, we did not detect a time-dependent trend in our study population throughout the observation period [2, 7]. Therefore, viral hepatitis remains a main cause of ALF in Eastern Austria. DILI was relatively rare (accounting for 10% of our observed ALF cases) in our study population, as compared to other European studies reporting DILI as (suspected) ALF cause in 16–51% [15–18]. Interestingly, no case of acetaminophen-induced liver failure was reported in our single-center analysis, which does not reflect the situation in other European countries. Early management of acetaminophen overdosing in emergency outpatient wards, dispensing regulations of acetaminophen, and guidance for patients in licensed pharmacies might be a reason for the lack of acetaminophen-induced liver failure in our Eastern Austrian study cohort. We detected two cases of anticipated ALF admitted to our ICU with acetaminophen intoxication. However, those patients never fulfilled the EASL criteria for ALF and were therefore excluded from this study [2].

The overall ICU survival among our study population was 61%, which is comparable with other European studies [9, 15, 18, 19]. More importantly, HU-LTx had a significant positive impact on ICU survival, with patients who received a transplant showing a 28-day survival of 92% and a 6-month survival of 85%. These data are comparable with other published studies in Central Europe and underscore the long-term benefits of timely transplantation in ALF patients [2, 9, 19]. In our study, the median time from ICU admission to transplantation was 3 days, emphasizing well-functioning multidisciplinary work between transplant surgeons, hepatologists, and intensivists. Given the rapid progression of ALF in this patient population, early identification and early consideration for HU-LTx are of utmost importance in optimizing patient outcomes [7, 20].

When determining transplant eligibility in ALF patients, comprehensive multidisciplinary evaluation is crucial for optimal organ allocation and long-term survival [21]. In Austria, three centers, Vienna, Graz, and Innsbruck, are qualified to perform HU-LTx. From 2012 to 2024, 13 ALF patients received HU-LTx at the Medical University of Graz. The Medical University of Innsbruck conducted 41 HU-LTx procedures for ALF patients. In our study population, 42% of all patients (*n* = 13) were transplanted. Other studies reported higher frequencies of HU-LTx, although they only included patients listed for HU-LTx in their analysis [16, 18, 22]. Patients were mainly excluded from HU-LTx based on reasons like malignancy or IVDA, known as relevant contraindications [23]. In our ALF cohort, 5 patients (16%) were listed but died before potential transplantation, which is comparable to the 16% in a US registry [22]. Of those 5 patients who died before potential transplantation, 3 patients had hyperacute and 2 acute onset ALF. Additionally, 3 of those 5 patients were already admitted to our ICU with overt HE of grades 3 to 4, indicating rapid clinical deterioration and disease progression. All 5 patients were listed for HU-LTx as soon as possible but subsequently died within 2–5 days due to rapidly progressive multiorgan failure. Although patients in our cohort were generally transplanted rather early (a median time from admission to transplantation of 3 days), 3 of the discussed patients stayed at the ICU for 4 to 5 days without suitable organ offer. The remaining 2 patients died within 2 days. Altogether, late recognition of ALF, late transfer to the ICU, rapid disease progression, and prolonged waiting time for a suitable donor organ might all be possible causes for death before transplantation in these cases.

Different extracorporeal therapies were used in our study population to support liver function. However, data on liver support systems are still conflicting and heavily debated. Except for plasma exchange (PE), no positive results on survival were reported among extracorporeal liver support systems in ALF patients [2]. However, the positive effects of PE reported by Larsen et al. were predominantly seen in patients with no option for HU-LTx and those not listed due to comorbidities [24]. This patient population represented a minority in our study. A more recent study on PE in patients with ALF showed significant hemodynamic changes but no significant impact on overall survival [25]. Nine patients in our study cohort received CytoSorb therapy in addition to RRT. The decision for additional CytoSorb adsorber application was thereby always made by the treating physicians. The CytoSorb cartridges are easily applicable hemadsorption tools that impact bilirubin and further laboratory parameters among patients with chronic liver disease [26]. Nevertheless, data on CytoSorb as an extracorporeal liver support system in ALF patients are still scarce, and future prospective studies are warranted to investigate its value in ALF therapy [27, 28].

Our findings illustrate characteristic laboratory changes indicating a complex interplay of liver damage and dysfunction [2]. Elevated liver enzymes, as hallmark parameters for massive hepatocyte damage and necrosis, were significantly declining due to the exhaustion of the hepatocyte population. Constantly deranged coagulation parameters indicated impairment of liver synthesis. Improving INR and prothrombin time in ICU survivors reflected the state after HU-LTx. Despite not reaching statistical significance, there was a trend to constantly lower platelets among nonsurvivors, which might represent a potential marker for outcome among ALF patients [29, 30]. Ammonia levels were initially elevated but not extensively high, which might reflect early management of ammonia control. However, 64% of our patients were intubated due to HE. These data suggest that ammonia might not serve as a standalone surrogate parameter for HE, and clinical examination several times a day is still very important [14, 30]. In the case of overt HE, preventive therapies against hyperammonemia were administered according to EASL guidelines in order to prevent the development of brain edema, which represents a common cause of death in ALF patients [2, 7]. At our center, we mainly use clinical parameters (neurological examination at short time intervals) for the monitoring of intracerebral pressure. Although recommended by the EASL guidelines, invasive ICP monitoring is not routinely done. In case of suspected cerebral edema, a cranial computer tomography scan was conducted. However, we did not observe any case of brain edema in our patient cohort.

Our study is one of the first studies reporting Austrian data on ALF patients admitted to the ICU; however, it has some limitations. First, due to the retrospective design of our study, missing information regarding further characterization of ALF cannot be excluded. Second, over the course of 12 years, we have only reported a relatively small ALF cohort of 31 patients. ALF is indeed a rare disease and is primarily managed in specialized transplantation centers. We investigated all ALF patients admitted to the largest tertiary center in Eastern Austria and gathered the first epidemiologic data in Austria. Moreover, we only analyzed the patient’s ICU stay before and after a potential HU-LTx. We did not collect data on post-ICU treatment. However, the primary aim was to create a homogenous group focused on ALF management during ICU stay. Because of these limitations, we urge for a prospective multicenter registry on ALF patients in Austria in order to accomplish a more precise characterization of ALF patients. This registry may serve as a foundation for future prospective studies.

## Conclusion

Our single-center study on acute liver failure (ALF) patients admitted to the intensive care unit (ICU) in Eastern Austria found viral hepatitis (26%), autoimmune hepatitis (AIH; 16%), Wilson’s disease (13%), and drug-induced liver injury (DILI; 10%) as the most frequent causes. We did not identify any confirmed cases of acetaminophen-induced liver failure. However, toxins (3%), vascular (3%), and malignant diseases (3%) were relatively rare causes of ALF. This study highlights the complexity of ALF management at the ICU, emphasizing the critical role of high-urgency liver transplantation (HU-LTx) in improving survival. In total, 16% of our study population died before potential transplantation. Due to the rarity of ALF and its significant implications for patient outcomes, our data support the establishment of a prospective multicenter registry in Austria to gather an advanced understanding of the underlying ALF causes, ICU management, and unbiased outcome data for this rare patient population exhibiting a high short-term mortality if not transplanted.

## Supplementary Information

Supplementary tables and figures include further demographic details and laboratory data of ALF patients investigated in this study.

## Data Availability

Data are available from the corresponding author upon reasonable request.

## Abbreviations

AIH: Autoimmune hepatitis
ALF: Acute liver failure
ALI: Acute liver injury
AP: Alkaline phosphatase
CRP: C‑reactive protein
DILI: Drug-induced liver injury
EASL: European Association for the Study of the Liver
EBV: Epstein–Barr virus
HAV: Hepatitis A virus
HBV: Hepatitis B virus
HE: Hepatic encephalopathy
HLH: Hemophagocytic lymphohistiocytosis
HU-LTx: High-urgency liver transplantation
ICU: Intensive care unit
IMV: Invasive mechanical ventilation
INR: International normalized ratio
IQR: Interquartile range
IVDA: Intravenous drug abuse
LOS: Length of stay
RRT: Renal replacement therapy
SAPS II: Simplified acute physiology score II
SD: Standard deviation
SOFA: Sequential organ failure assessment
ULN: Upper limit of normal
WBC: White blood cell count
