# Supplementary material for: Acute liver failure in patients admitted to the intensive care unit—a Viennese retrospective single-center analysis
Source: Wien Klin Wochenschr. 2025 May 26;137(21-22):720–9. doi: 10.1007/s00508-025-02539-1 (PMC12592254; doi:10.1007/s00508-025-02539-1)
Supplement: Supplementary file 1 — Supplementary tables and figures include further demographic details and laboratory data of ALF patients investigated in this study. [file 508_2025_2539_MOESM1_ESM.docx]

**Supplementary Material**

**Acute liver failure in patients admitted to the Intensive Care Unit - a Viennese retrospective single center analysis**

Haselwanter Patrick^1^, Fairfield Seanna^1^, Riedl-Wewalka Marlene^1^, Schmid Monika^1^, Stättermayer Albert Friedrich^1^, Reiberger Thomas^1^, Trauner Michael^1^, Zauner Christian^1^, Schneeweiss-Gleixner Mathias^1^

^1^ Department of Medicine III, Division of Gastroenterology and Hepatology, Intensive Care Unit 13H1, Medical University of Vienna, Vienna, Austria

Supplemental Tables

**Supplemental Table S1:** Comparison of baseline characteristics between HU-LTx and no HU-LTx.

|  | **total population** | **HU-LTx** | **without HU-LTx** | ***p-value*** |
| --- | --- | --- | --- | --- |
| **N (%)** | 31 (100) | 13 (42) | 18 (58) |  |
| **Age, median (IQR)** | 44 (32-56) | 38 (29-44) | 56 (41-59) | **0.0064** |
| **Age distribution** |  |  |  |  |
| **18 – 24, n (%)** | 3 (10) | 2 (15) | 1 (5.5) | 0.3610 |
| **25 – 34, n (%)** | 7 (23) | 4 (31) | 3 (17) | 0.3540 |
| **35 – 44, n (%)** | 6 (19) | 5 (38) | 1 (5.5) | **0.0221** |
| **45 – 54, n (%)** | 5 (16) | 2 (15) | 3 (17) | 0.9237 |
| **55 – 64, n (%)** | 9 (29) | 0 | 9 (50) | **0.0024** |
| **> 64, n (%)** | 1 (3) | 0 | 1 (5.5) | 0.3876 |
| **male/female** | 11/20 | 4/9 | 7/11 | 0.6410 |
| **BMI, median (IQR)** | 25.7 (22.2-28.6) | 26.36 (23.5-30.5) | 25.52 (22.1-28.2) | 0.7415 |
| **Etiology of ALF** |  |  |  |  |
| **Viral, n (%)** | 8 (26) | 3 (23) | 5 (28) | 0.7678 |
| **HBV, n (%)** | 6 (75) | 2 (67) | 4 (80) | 0.6344 |
| **HAV, n (%)** | 1 (12.5) | 1 (33) | 0 | 0.2316 |
| **EBV, n (%)** | 1 (12.5) | 0 | 1 (20) | 0.3876 |
| **Unknown, n (%)** | 8 (23) | 4 (31) | 4 (22) | 0.5915 |
| **AIH, n (%)** | 5 (16) | 3 (23) | 2 (11) | 0.3714 |
| **DILI, n (%)** | 3 (10) | 0 | 3 (17) | 0.1214 |
| **Wilson Disease, n (%)** | 4 (13) | 3 (23) | 1 (5.5) | 0.1510 |
| **Toxins, n (%)** | 1 (3) | 0 | 1 (5.5) | 0.3876 |
| **Vascular, n (%)** | 1 (3) | 0 | 1 (5.5) | 0.3876 |
| **Malignant, n (%)** | 1 (3) | 0 | 1 (5.5) | 0.3876 |
| **ICU LOS (days), median (IQR)** | 12 (4-21) | 17 (13-24) | 5 (4-12) | **0.0019** |
| **Vasopressor therapy, n (%)** | 21 (68) | 10 (77) | 11 (61) | 0.3527 |
| **IMV, n (%)** | 26 (83) | 13 (100) | 13 (72) | **0.0379** |
| **Reason for IMV** |  |  |  |  |
| **HE, n (%)** | 16 (64) | 6 (46) | 10 (77) | 0.6052 |
| **HU-LTx, n (%)** | 7 (28) | 7 (54) | 0 | **0.0004** |
| **Respiratory failure, n (%)** | 3 (12) | 0 | 3 (23) | 0.1214 |
| **Length of IMV (days), median (IQR)** | 2 (1-6) | 2 (1-5) | 5.5 (1-10.5) | 0.7374 |
| **ICU adm. to IMV (days), median (IQR)** | 1 (0-3) | 2 (1-4) | 0.5 (0-2.5) | 0.0386 |
| **EC therapy*, n (%)** | 24 (77) | 10 (77) | 14 (78) | 0.9552 |
| **RRT, n (%)** | 23 (74) | 10 (77) | 13 (72) | 0.7678 |
| **ECMO, n (%)** | 2 (6) | 1 (8) | 1 (7) | 0.8111 |
| **PE, n (%)** | 2 (6) | 1 (8) | 1 (7) | 0.8111 |
| **MARS, n (%)** | 5 (16) | 1 (8) | 4 (22) | 0.2777 |
| **CytoSorb Adsorbers, n (%)** | 9 (29) | 6 (46) | 3 (17) | 0.3236 |
| **SAPS II**, mean±SD** | 40.97±14.84 | 34.38±9.36 | 45.72±16.43 | **0.0333** |
| **SOFA**, mean±SD** | 10.55±4.56 | 9.85±4.74 | 11.06±4.49 | 0.4755 |
| **ICU survival, n (%)** | 19 (61) | 11 (85) | 8 (44) | **0.0234** |
| **28-day survival, n (%)** | 18 (58) | 12 (92) | 6 (33) | **0.0010** |
| **3-month survival, n (%)** | 17 (55) | 11 (85) | 6 (33) | **0.0046** |
| **6-month survival, n (%)** | 17 (55) | 11 (85) | 6 (33) | **0.0046** |

*Abbreviations: Adm., admission; AIH, autoimmune hepatitis; ALF, acute liver failure; BMI, body mass index; EBV, Epstein-Barr virus; EC, extracorporeal; ECMO,* *extracorporeal membrane oxygenation; HAV, Hepatitis A virus; HBV, Hepatitis B virus; HE, hepatic encephalopathy; HU-LTx, high-urgency liver transplantation; ICU LOS, intensive care unit length of stay; IMV, invasive mechanical ventilation; IQR, interquartile range; n, population size; PE, plasma exchange; RRT, renal replacement therapy; SAPS II, simplified acute physiology score II; SD, standard deviation SOFA, sequential organ failure assessment score.*

** EC Therapy including RRT, plasma exchange, liver-assist devices, ECMO; ** SAPS II and SOFA score were calculated within the first 24 hours after admission.*

**Supplemental Table S2:** Gender-specific comparison of baseline characteristics.

|  | **total population** | **female** | **male** | ***p-value*** |
| --- | --- | --- | --- | --- |
| **N (%)** | 31 (100) | 20 (65) | 11 (35) |  |
| **Age, median (IQR)** | 44 (32-56) | 43 (33-54) | 52 (36-58) | 0.5018 |
| **Age distribution** |  |  |  |  |
| **18 – 24, n (%)** | 3 (10) | 1 (5) | 2 (18) | 0.2349 |
| **25 – 34, n (%)** | 7 (23) | 6 (30) | 1 (9) | 0.1827 |
| **35 – 44, n (%)** | 6 (19) | 4 (20) | 2 (18) | 0.9024 |
| **45 – 54, n (%)** | 5 (16) | 4 (20) | 1 (9) | 0.4294 |
| **55 – 64, n (%)** | 9 (29) | 4 (20) | 5 (45) | 0.1351 |
| **> 64, n (%)** | 1 (3) | 1 (5) | 0 | 0.4509 |
| **BMI, median (IQR)** | 25.7 (22.2-28.6) | 23.4 (21.8-26.5) | 28.6 (25.7-31.6) | 0.4447 |
| **Etiology of ALF** |  |  |  |  |
| **Viral, n (%)** | 8 (26) | 4 (20) | 4 (36) | 0.3191 |
| **HBV, n (%)** | 6 (75) | 3 (75) | 3 (75) | 0.4079 |
| **HAV, n (%)** | 1 (12.5) | 0 | 1 (25) | 0.1704 |
| **EBV, n (%)** | 1 (12.5) | 1 (25) | 0 | 0.4509 |
| **Unknown, n (%)** | 8 (26) | 7 (35) | 1 (9) | 0.1147 |
| **AIH, n (%)** | 5 (16) | 5 (25) | 0 | 0.0701 |
| **DILI, n (%)** | 3 (10) | 2 (10) | 1 (9) | 0.9347 |
| **Wilson Disease, n (%)** | 4 (13) | 1 (5) | 3 (27) | 0.0767 |
| **Toxins, n (%)** | 1 (3) | 1 (5) | 0 | 0.4509 |
| **Vascular, n (%)** | 1 (3) | 0 | 1 (9) | 0.1704 |
| **Malignant, n (%)** | 1 (3) | 0 | 1 (9) | 0.1704 |
| **ICU LOS (days), median (IQR)** | 12 (4-21) | 13 (5.5-21.75) | 8 (4.5-16) | 0.4761 |
| **Vasopressor therapy, n (%)** | 21 (68) | 15 (75) | 6 (55) | 0.2437 |
| **IMV, n (%)** | 26 (83) | 18 (90) | 8 (73) | 0.2109 |
| **Reason for IMV** |  |  |  |  |
| **HE, n (%)** | 16 (64) | 13 (72) | 3 (38) | 0.0443 |
| **HU-LTx, n (%)** | 7 (28) | 3 (17) | 4 (50) | 0.1734 |
| **Respiratory failure, n (%)** | 3 (12) | 2 (11) | 1 (13) | 0.9347 |
| **Length of IMV (days), median (IQR)** | 2 (1-6) | 5.5 (2-8.5) | 1 (1-3) | **0.0395** |
| **ICU adm. to IMV (days), median (IQR)** | 1 (0-3) | 1 (0-2) | 2.5 (1.75-3.25) | 0.1772 |
| **EC therapy*, n (%)** | 24 (77) | 18 (90) | 6 (55) | **0.0238** |
| **RRT, n (%)** | 23 (74) | 18 (90) | 5 (45) | **0.0066** |
| **ECMO, n (%)** | 2 (6) | 1 (6) | 1 (9) | 0.6573 |
| **PE, n (%)** | 2 (6) | 1 (6) | 1 (9) | 0.6573 |
| **MARS, n (%)** | 5 (16) | 2 (11) | 3 (27) | 0.2109 |
| **CytoSorb Adsorbers, n (%)** | 9 (29) | 7 (35) | 2 (16) | 0.9756 |
| **SAPS II**, mean±SD** | 40.97±14.84 | 41.65±17.12 | 39.73±10.06 | 0.7363 |
| **SOFA**, mean±SD** | 10.55±4.56 | 10.80±5.03 | 10.09±3.73 | 0.6859 |
| **Liver transplant, n (%)** | 13 (42) | 9 (45) | 4 (36) | 0.6410 |
| **ICU survival, n (%)** | 19 (61) | 12 (60) | 7 (63) | 0.8423 |
| **28-day survival, n (%)** | 18 (58) | 11 (55) | 7 (63) | 0.6410 |
| **3-month survival, n (%)** | 17 (55) | 10 (50) | 7 (63) | 0.4654 |
| **6-month survival, n (%)** | 17 (55) | 10 (50) | 7 (63) | 0.4654 |

*Abbreviations: Adm., admission; AIH, autoimmune hepatitis; ALF, acute liver failure; BMI, body mass index; EBV, Epstein-Barr virus; EC, extracorporeal; ECMO,* *extracorporeal membrane oxygenation; HAV, Hepatitis A virus; HBV, Hepatitis B virus; HE, hepatic encephalopathy; HU-LTx, high-urgency liver transplantation; ICU LOS, intensive care unit length of stay; IMV, invasive mechanical ventilation; IQR, interquartile range; n, population size; PE, plasma exchange; RRT, renal replacement therapy; SAPS II, simplified acute physiology score II; SD, standard deviation SOFA, sequential organ failure assessment score.*

** EC Therapy including RRT, plasma exchange, liver-assist devices, ECMO; ** SAPS II and SOFA score were calculated within the first 24 hours after admission.*

**Supplemental Table S3:** Trajectory of laboratory parameters.

| **Laboratory parameters** | **total population** | ***p-value*** | **ICU survivors** | ***p-value*** |  | **nonsurvivors** | ***p-value*** |
| --- | --- | --- | --- | --- | --- | --- | --- |
| **Bilirubin n (%)** | 31 (100) |  | 19 (100) |  |  | 12 (100) |  |
| *median difference after 24h in mg/dL* | **-1.50** | **0.0164** | **-1.80** | **0.0204** |  | -0.550 | 0.4238 |
| *median difference to discharge in mg/dL* | **-3.17** | **0.0042** | **-9.60** | **<0.0001** |  | 2.36 | 0.4575 |
| **AP n (%)** | 31 (100) |  | 19 (100) |  |  | 12 (100) |  |
| *median difference after 24h in U/L* | **-21.0** | **<0.0001** | **-17.0** | **0.0004** |  | -74.0 | 0.1294 |
| *median difference to discharge in U/L* | 29.0 | 0.4413 | **49.0** | **0.0085** |  | **-65.5** | **0.0020** |
| **γ-GT n (%)** | 31 (100) |  | 19 (100) |  |  | 12 (100) |  |
| *median difference after 24h in U/L* | **-25.0** | **<0.0001** | **-17.0** | **<0.0001** |  | **-38.5** | **0.0005** |
| *median difference to discharge in U/L* | -9.0 | 0.3493 | **90.0** | **0.0181** |  | -45.0 | 0.1050 |
| **ASAT n (%)** | 31 (100) |  | 19 (100) |  |  | 12 (100) |  |
| *median difference after 24h in U/L* | **-214** | **<0.0001** | **-196** | **0.0009** |  | **-417** | **0.0024** |
| *median difference to discharge in U/L* | **-626** | **<0.0001** | **-626** | **<0.0001** |  | **-639** | **0.0269** |
| **ALAT n (%)** | 31 (100) |  | 19 (100) |  |  | 12 (100) |  |
| *median difference after 24h in U/L* | **-333** | **<0.0001** | **-282** | **<0.0001** |  | **-380** | **0.0024** |
| *median difference to discharge in U/L* | **-977** | **<0.0001** | **-977** | **0.0003** |  | **-931** | **0.0068** |
| **Albumin n (%)** | 31 (100) |  | 19 (100) |  |  | 12 (100) |  |
| *median difference after 24h in g/dL* | **-2.48** | **0.0236** | -0.800 | 0.0506 |  | -2.20 | 0.2036 |
| *median difference to discharge in g/dL* | **-2.60** | **0.0045** | **-3.30** | **0.0145** |  | -2.30 | 0.1099 |
| **Ammonia n (%)** | 31 (100) |  | 19 (100) |  |  | 12 (100) |  |
| *median difference after 24h in µg/dL* | 5.30 | 0.5390 | 3.00 | >0.9999 |  | 13.1 | 0.4238 |
| *median difference to discharge in µg/dL* | -10.2 | 0.4074 | -10.9 | 0.5153 |  | -8.15 | 0.7754 |
| **CRP n (%)** | 31 (100) |  | 19 (100) |  |  | 12 (100) |  |
| *median difference after 24h in mg/dL* | **-0.14** | **0.0091** | -0.090 | 0.0974 |  | **-0.155** | **0.0332** |
| *median difference to discharge in mg/dL* | 0.85 | 0.0961 | 0.950 | 0.1564 |  | -0.175 | 0.3652 |
| **INR n (%)** | 31 (100) |  | 19 (100) |  |  | 12 (100) |  |
| *median difference after 24h* | **-0.99** | **0.0091** | 0.00 | 0.9748 |  | 0.20 | 0.4575 |
| *median difference to discharge* | 0.16 | 0.4738 | **-2.00** | **0.0010** |  | -0.05 | 0.9658 |
| **Prothrombin time n (%)** | 31 (100) |  | 19 (100) |  |  | 12 (100) |  |
| *median difference after 24h in %* | -1.00 | 0.8708 | 0.00 | 0.9908 |  | -2.50 | 0.8945 |
| *median difference to discharge in %* | **21.0** | **0.0002** | **39.0** | **<0.0001** |  | -1.50 | 0.9121 |
| **Fibrinogen n (%)** | 31 (100) |  | 19 (100) |  |  | 12 (100) |  |
| *median decrease after 24h in mg/dL* | 0.00 | 0.8106 | 19.0 | 0.2837 |  | -26.5 | 0.0645 |
| *median decrease to discharge in mg/dL* | **58.0** | **0.0050** | **256** | **0.0002** |  | -30.5 | 0.2578 |
| **D-Dimer n (%)** | 19 (61) |  | 9 (47) |  |  | 10 (83) |  |
| *median difference after 24h in mg/dL* | 0.30 | 0.0680 | 0.200 | 0.3370 |  | 2.15 | 0.1055 |
| *median difference to discharge in mg/dL* | 3.87 | 0.2579 | -0.100 | 0.9102 |  | 6.20 | 0.1641 |
| **Haemoglobin n (%)** | 31 (100) |  | 19 (100) |  |  | 12 (100) |  |
| *median difference after 24h in g/dL* | **-1.00** | **0.0021** | **-1.20** | **0.0048** |  | -0.750 | 0.1763 |
| *median difference to discharge in g/dL* | **-1.50** | **0.0065** | **-1.20** | **0.0457** |  | -1.90 | 0.0801 |
| **WBC n (%)** | 31 (100) |  | 19 (100) |  |  | 12 (100) |  |
| *median difference after 24h in G/L* | **-2.03** | **0.0157** | -2.03 | 0.0663 |  | -2.06 | 0.3804 |
| *median difference to discharge in G/L* | -2.07 | 0.6394 | -2.23 | 0.3124 |  | -0.350 | 0.6772 |
| **Platelets n (%)** | 31 (100) |  | 19 (100) |  |  | 12 (100) |  |
| *median difference after 24h in G/L* | **-35.0** | **0.0002** | **-42.0** | **0.0032** |  | -34.5 | 0.0640 |
| *median difference to discharge in G/L* | -46.0 | 0.2449 | 0.00 | 0.9661 |  | **-58.5** | **0.0342** |

*Abbreviations: ALAT, alanine aminotransferase; AP, alkaline phosphatase; ASAT, aspartate aminotransferase; CRP, c-reactive protein; γ-GT, gamma-glutamyl transferase; INR, international normalized ratio; kU/L, kilo Units per Liter; mg/dL, milligrams per Deciliter; U/L, Units per Liter; WBC, white blood count; g/dL, grams per Deciliter; G/L, Giga per Liter.*

Median differences of laboratory parameters from ICU admission to 24 hours and ICU admission to ICU discharge. In the nonsurvivors group discharge values are depicted last laboratory values before death. Significant median differences and p-value were marked as bold if the p-value was <0.05. Values of D-Dimer at 24 hours or at discharge were lacking in occasional patients.

Supplemental Figures

**Supplemental Figure S1:**

**Figure Legend Supplemental Figure S1: Flowchart of the ALF study population.** Patients with acute liver injury or anticipated acute liver failure who never developed transaminases, INR, and HE, according to the EASL definition of ALF, were generally excluded from the study population. *Abbreviations: HE, hepatic encephalopathy; ICU, intensive care unit; INR, international normalized ratio; TA, transaminases.*

**Supplemental Figure S2a:** Proportional representation of age in total population, ICU survivors and nonsurvivors.

**Figure Legend Supplemental Figure S2a: Proportional representation of age in total population, ICU survivors and nonsurvivors.** Age is given in different colors in the subgroups 18 to 24, 25 to 34, 35 to 44, 45 to 54, 55 to 64, and above 65 years. Relative frequencies of age in the total population, ICU survivors, and nonsurvivors are depicted in Table 1. *Abbreviations: ICU, intensive care unit.*

**Supplemental Figure S2b:** Proportional representation of ALF subclassifications in total population, ICU survivors and nonsurvivors.

**Figure Legend Supplemental Figure S2b: Proportional representation of ALF onset in total population, survivors and nonsurvivors.** Onset was defined as development from jaundice to hepatic encephalopathy. Relative frequencies of hyperacute (0 to 7 days of onset), acute (8 to 28 days of onset), and subacute (28 days to 12 weeks of onset) liver failure in the total population, ICU survivors, and nonsurvivors are given in each pie chart. *Abbreviations: ICU, intensive care unit.*

**Supplemental Figure S3:**

**Figure Legend** **Supplemental Figure S3:** Median values with IQR of Bilirubin, AP, γ-GT, ASAT, ALAT, Albumin, Ammonia and CRP were displayed at admission, 24 hours after ICU admission, and before discharge or death displayed as dots with error bars. Median changes in ICU survivors were marked as a light blue line, a dark blue line in nonsurvivors, and a bold black line in the total population. *Abbreviations: ALAT, alanine aminotransferase; AP, alkaline phosphatase; ASAT, aspartate aminotransferase, CRP, c-reactive protein*

**Supplemental Figure S4:**

**Figure Legend Supplemental Figure S4:** Median values with IQR of INR, Prothrombin time, Fibrinogen, D-dimer and hematological parameter (Hemoglobin, WBC, Platelets) were displayed at admission, 24 hours after ICU admission, and before discharge or death as dots with error bars. Median changes in ICU survivors were marked as a light blue line, a dark blue line in nonsurvivors, and a bold black line in the total population. *Abbreviations: INR, international normalized ratio, WBC, white blood count*
